# The Smallest Capsid Protein Mediates Binding of the Essential Tegument Protein pp150 to Stabilize DNA-Containing Capsids in Human Cytomegalovirus

**DOI:** 10.1371/journal.ppat.1003525

**Published:** 2013-08-15

**Authors:** Xinghong Dai, Xuekui Yu, Hao Gong, Xiaohong Jiang, Gerrado Abenes, Hongrong Liu, Sakar Shivakoti, William J. Britt, Hua Zhu, Fenyong Liu, Z. Hong Zhou

**Affiliations:** 1 Department of Microbiology, Immunology and Molecular Genetics, University of California, Los Angeles, Los Angeles, California, United States of America; 2 The California NanoSystems Institute (CNSI), University of California, Los Angeles, Los Angeles, California, United States of America; 3 Division of Infectious Diseases, School of Public Health, University of California, Berkeley, Berkeley, California, United States of America; 4 Department of Pediatrics, School of Medicine, University of Alabama at Birmingham, Birmingham, Alabama, United States of America; 5 Department of Microbiology and Molecular Genetics, University of Medicine and Dentistry of New Jersey, Newark, New Jersey, United States of America; Johns Hopkins School of Medicine, United States of America

## Abstract

Human cytomegalovirus (HCMV) is a ubiquitous herpesvirus that causes birth defects in newborns and life-threatening complications in immunocompromised individuals. Among all human herpesviruses, HCMV contains a much larger dsDNA genome within a similarly-sized capsid compared to the others, and it was proposed to require pp150, a tegument protein only found in cytomegaloviruses, to stabilize its genome-containing capsid. However, little is known about how pp150 interacts with the underlying capsid. Moreover, the smallest capsid protein (SCP), while dispensable in herpes simplex virus type 1, was shown to play essential, yet undefined, role in HCMV infection. Here, by cryo electron microscopy (cryoEM), we determine three-dimensional structures of HCMV capsid (no pp150) and virion (with pp150) at sub-nanometer resolution. Comparison of these two structures reveals that each pp150 tegument density is composed of two helix bundles connected by a long central helix. Correlation between the resolved helices and sequence-based secondary structure prediction maps the tegument density to the N-terminal half of pp150. The structures also show that SCP mediates interactions between the capsid and pp150 at the upper helix bundle of pp150. Consistent with this structural observation, ribozyme inhibition of SCP expression in HCMV-infected cells impairs the formation of DNA-containing viral particles and reduces viral yield by 10,000 fold. By cryoEM reconstruction of the resulting “SCP-deficient” viral particles, we further demonstrate that SCP is required for pp150 functionally binding to the capsid. Together, our structural and biochemical results point to a mechanism whereby SCP recruits pp150 to stabilize genome-containing capsid for the production of infectious HCMV virion.

## Author Summary

Human cytomegalovirus (HCMV) causes birth defects in newborns and life-threatening complications in immunocompromised individuals, such as AIDS patients and organ transplant recipients. The smallest capsid protein (SCP) – only 8 kDa molecular mass as compared to the 155 kDa major capsid protein – has been demonstrated to be essential for HCMV growth, but is dispensable in herpes simplex virus type 1. These seemingly contradictory observations have been a paradox. Here, we solve this paradox by high resolution cryo electron microscopy (cryoEM), in conjunction with functional studies using ribozyme inhibition. Our structural comparisons of HCMV virion and capsid reveal molecular interactions at the secondary structure level and suggest that SCP might contribute to capsid binding of pp150, an essential, cytomegalovirus-specific tegument protein. SCP-deficient particles generated by ribozyme inhibition of SCP-expression in HCMV-infected cells show no pp150 tegument density, demonstrating that SCP is required for the functional binding of pp150 to the capsid. Our results suggest that SCP recruits pp150 to stabilize the HCMV nucleocapsid to enable encapsidation of the genome, which is more densely packaged in HCMV than in other herpesviruses. Overall, this study not only resolves the above paradox, but also illustrates the *passive acquisition* of a new, essential function by SCP in the production of infectious HCMV virions.

## Introduction

Human cytomegalovirus (HCMV), the prototype of betaherpesvirus subfamily of the *Herpesviridae*, is a leading viral cause of birth abnormalities and a major life-threatening pathogen in AIDS and organ transplant patients [1]. HCMV virion shares a common architecture with other herpesviruses and consists of a polymorphic envelope, a tegument compartment and an icosahedral nucleocapsid enclosing a linear dsDNA genome. The HCMV genome is the largest amongst that of all human herpesviruses, and encodes a remarkable number of conserved proteins, as well as unique envelope and tegument proteins that lack homologs in alpha- or gammaherpesviruses [2]. The HCMV capsid shell, similar to those of herpes simplex virus type 1 (HSV-1) and Kaposi's sarcoma-associated herpesvirus (KSHV), is composed of four major proteins: the major capsid protein (MCP; encoded by UL86) [3], the minor capsid protein (mCP; encoded by UL85), the mCP binding protein (mC-BP; encoded by UL46) [4], and the smallest capsid protein (SCP; encoded by UL48.5) [5], [6]. All herpesvirus capsids studied to date share a T = 16 icosahedral assembly with pentons (MCP pentomers), hexons (hexamers of MCP), connecting triplexes (heterotrimers of two mCP and one mC-BP), and SCP attached to the tip of each MCP [7], [8], [9], [10], [11]. While the other three capsid structural proteins are conserved, SCP is very divergent in size, amino acid sequence and function among different herpesviruses. In HCMV, SCP was shown to be essential for virus growth [12], but its function is still unknown.

CryoEM reconstruction also revealed different patterns of association between capsid and overlying tegument proteins in CMV and HSV. In HCMV, a layer of highly organized filamentous density of tegument proteins is attached to the pentons, hexons and triplexes of the underlying nucleocapsid [13]. The three-dimensional (3D) reconstruction of the simian cytomegalovirus (SCMV) capsid isolated from the cytoplasm of infected cells also revealed tegument proteins attached to the capsid [8], similar to HCMV. In contrast, HSV-1 ordered tegument proteins only bind pentons and those triplexes surrounding pentons [14], [15], [16]. These observations indicate that viral proteins overlying the conserved capsid, such as tegument and envelope proteins, have evolved to have virus-specific structural and functional roles. Recently, biochemical and structural studies have assigned pp150 to the ordered filamentous tegument densities of CMV virion and suggested its function in stabilizing the dsDNA-filled C capsid [17], [18], but more structural details are needed to fully understand the molecular interactions between pp150 and capsid proteins.

Here, we report the 3D structures of HCMV capsid and intact HCMV virion at 6 Å and 9 Å resolution, respectively (Figure S1). Comparison of the capsid and virion structures reveals, at a secondary-structure level, that SCP mediates interactions between the capsid and tegument protein pp150. By constructing a ribozyme that inhibits SCP expression in HCMV-infected cells, and cryoEM reconstruction of the resulting “SCP-deficient” viral particles, we further demonstrate that SCP is required for pp150 binding to capsid and its absence results in only viral particles devoid of the DNA genome, thus revealing why SCP is essential for HCMV infection.

## Results

### CryoEM structural determination

**Figure 1:**
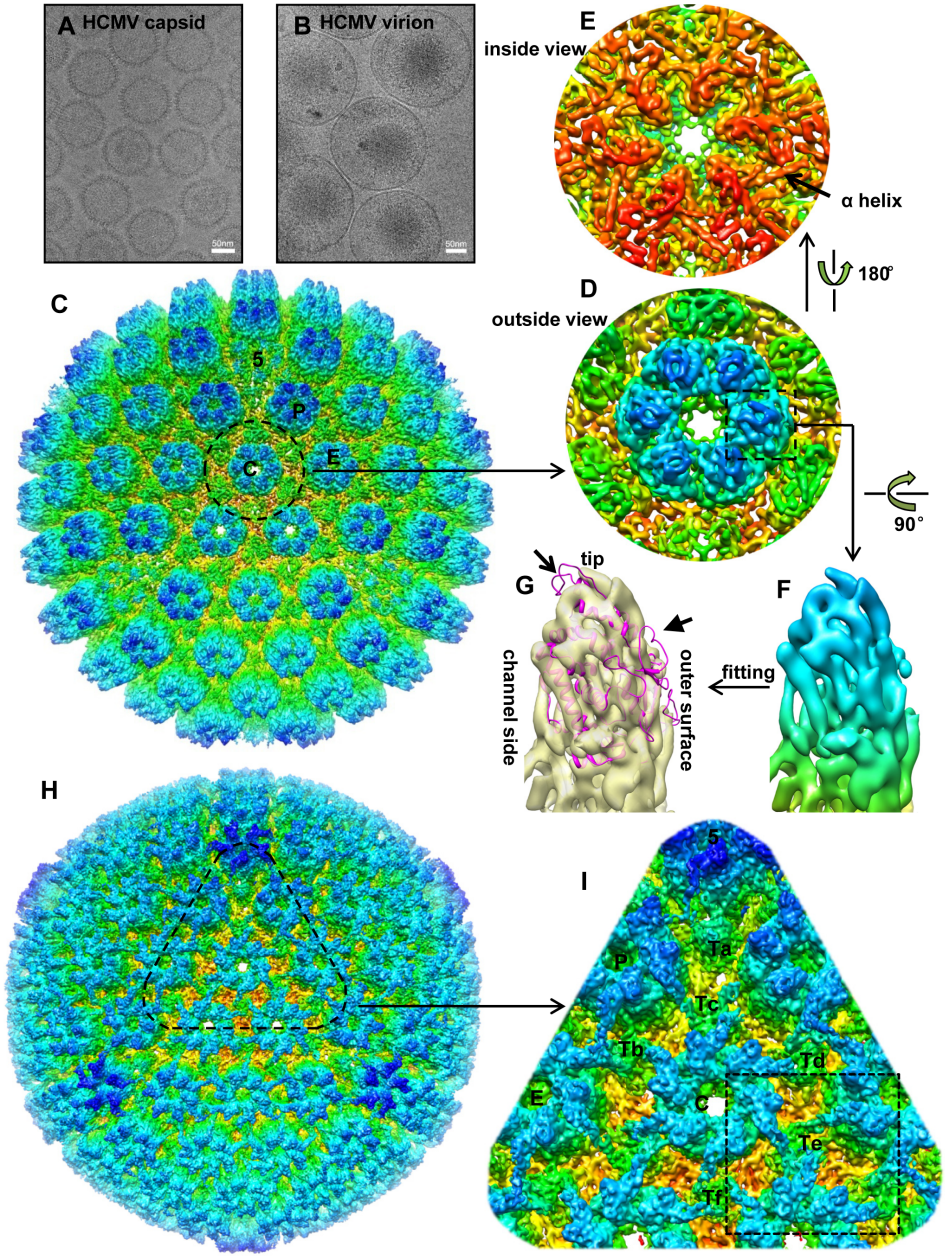
Comparison of 3D reconstructions of the HCMV capsid and virion. (A, B) CryoEM images of HCMV capsid (A) and virion (B). (C) Radially colored surface representation of the 3D reconstruction of the capsid at 6 Å resolution viewed along a 3-fold axis. Capsomers in an asymmetric unit, including a penton and three hexons, are labeled as “5”, “C”, “P” and “E”, respectively, as in the nomenclature of [39]. (D, E) Close-up views of the C hexon demarked in the capsid reconstruction, viewed from outside (D) or inside (E) of the capsid. An α-helix with typical sausage shape is denoted in (E). (F, G) MCPud. The density map of the MCPud denoted by the square in (D) was extracted and radially colored (F). In (G), the same MCPud is shown in semi-transparent yellow and superimposed with the HSV-1 MCPud atomic model (magenta ribbon). Note all helices match in the two structures but the loops at the tip (arrow) and at the outer surface (arrowhead) of HSV-1 MCPud do not fit the cryoEM density map of HCMV MCPud, suggesting possible structural differences. (H) Radially colored surface representation of the 3D reconstruction of the HCMV virion viewed along a 3-fold axis. (I) Zoom-in view of the area denoted in (H). Structural components in an asymmetric unit are labeled, including a penton (“5”), three hexons (“C”, “P” and “E”), and six triplexes (“Ta”, “Tb”, “Tc”, “Td”, “Te” and “Tf”). Dashed square demarcates a region encompassing Te that is segmented out for averaging with similar regions around Tb and Td (see text and Figure 2).

Due to the large size of CMV particles and the difficulties to purify them, resolutions of previous cryoEM reconstructions were limited [8], [10], [13], [19]. In this study, highly purified HCMV capsid (no tegument, Figure 1A) and the intact virion (containing tegument proteins, Figure 1B) were obtained and imaged in a 300 kV Titan Krios high-resolution electron microscope. 3D reconstructions of HCMV capsid and virion were obtained at 6 Å and 9 Å resolution, respectively (Figure S1). The improved structures are a result of an exhaustive effort of processing more than 37,000 capsid images and 56,000 virion images. At these sub-nanometer resolutions, secondary structural elements, particularly α-helices, can be identified, as exemplified by the close-up views of a hexon in the capsid reconstruction (Figure 1C–E). Molecular boundaries can be established, allowing us to describe the interactions between the ordered tegument proteins and capsid proteins at secondary-structure level for the first time.

### Comparisons of 3D reconstructions of the capsid and the virion

The 6 Å reconstruction of the HCMV capsid reveals the molecular boundaries among the 150 hexons, 12 pentons and 320 triplexes in the T = 16 icosahedral particle (Figure 1C), allowing identification of individual molecules. In particular, the upper domain of an MCP monomer was extracted from a central hexon (Figure 1F) and superimposed with the crystal structure of the HSV-1 MCP upper domain (MCPud) (PDB accession code 1N07) (Figure 1G) [20]. Except for very minor differences at the tip and the outer surface of the subunits (arrow and arrowhead in Figure 1G), excellent match of all the α-helices between HCMV and HSV-1 MCPuds is observed, indicating that the bulk of MCPud structure is conserved between the two viruses. This match also demonstrates the high quality of the map.

In addition, the fitting reveals that SCP molecules were lost in this highly purified capsid sample, probably due to the use of detergent during all purification steps (see Experimental Procedures). In gently prepared capsid preparations, SCP binds MCP at the upper domain as shown in previous capsid reconstructions we obtained [10], [19]. The absence of SCP in the capsid reconstruction provides the advantage to identify the molecular boundary between SCP and MCP in the virion reconstruction (see below).

**Figure 2:**
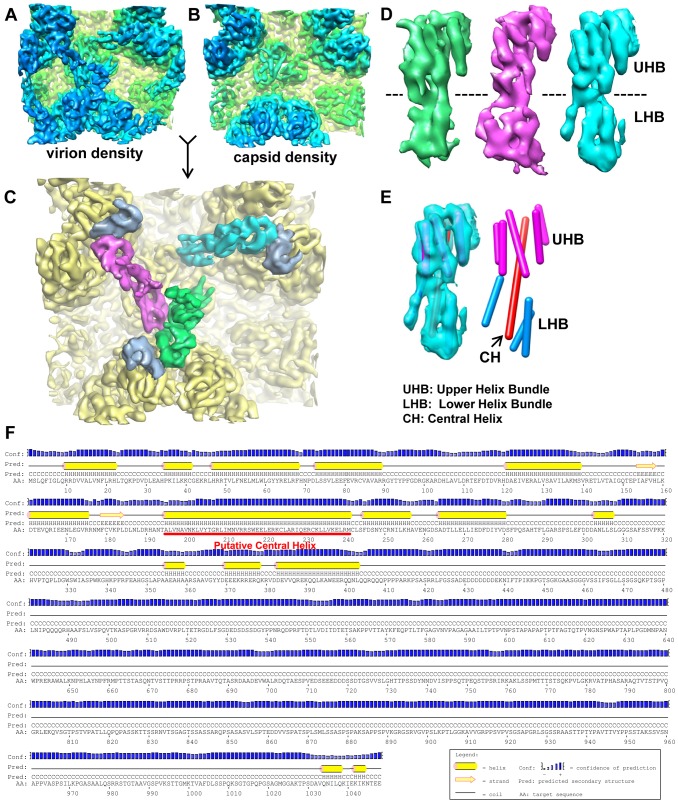
Structure of the capsid-interacting pp150 tegument protein. (A, B) Averaged density of the regions surrounding triplexes Tb, Td and Te (as marked by the dashed square in Figure 1I) from the virion (A) or capsid (B) reconstructions. The two density maps are colored the same as in Figure 1H or 1C. (C) SCP (light blue) and tegument (green, magenta and cyan) densities segmented from the virion densities of (A) superimposed on the capsid densities (yellow) of (B). (D) Views of the three tegument densities of (C) after alignment to each other. The most prominent feature is the sausage-shaped densities due to helices. The dashed line denotes the boundary of the upper helix bundle (UHB) and the lower helix bundle (LHB). (E) The cyan tegument density in (D) is shown semi-transparently and superimposed with cylinders representing helices. Helices in the upper helix bundle (magenta) and lower helix bundle (cyan) are connected by a 67 Å-long central helix (CH, red). (F) Secondary structure prediction of pp150 based on its amino acid sequence. The putative location for the long central helix identified in (E) is indicated by the red line.

The 9 Å reconstruction of HCMV virion shows a layer of filamentous tegument proteins bound to the capsid in an icosahedrally ordered fashion, like a net enclosing the entire capsid (Figure 1H). Three of these tegument densities sit on top of each triplex, forming a ‘group-of-three’, and extend to the top of the nearest subunits in the three surrounding capsomers. The location and appearance of these tegument densities are similar to those decorated by anti-pp150-antibodies [17], [18]. In each asymmetric unit of the herpesvirus capsid, there are six quasi-equivalent triplexes, Ta, Tb, Tc, Td, Te and Tf (Figure 1I), following the nomenclature of [21]. The group-of-three tegument densities on triplexes Tb, Td and Te are the most similar in structure. We averaged the densities within three cubes, each of which contains one of these group-of-three tegument densities (e.g., the region encompassing Te is outlined by the dashed square in Figure 1I) to improve the signal/noise ratio (Figure 2A). Helices in the tegument proteins can be resolved in the averaged density (as illustrated in Figure 2E). The same cubic regions from the capsid reconstruction were averaged for comparison (Figure 2B). This comparison allowed us to differentiate densities of MCP and triplexes from densities attributable to SCP and tegument proteins, and subsequently to segment out SCP and the tegument densities. The boundary between SCP and pp150 was established by referring to our pervious SCP-containing capsid reconstruction [19].

### Structure of the capsid-interacting tegument protein, pp150

The three tegument densities in the averaged group-of-three exhibit a high level of similarity and appear nearly identical when displayed side by side (Figure 2D). This structural similarity among the three tegument densities suggests that three copies of the same tegument protein or protein complex associate with each triplex, which differs from the situation in SCMV, where only two copies of tegument densities were interpreted to bind to each triplex [8]. In each tegument density of HCMV, we resolved two helix bundles, an upper one and a lower one, joined by a long central helix (∼67 Å in length). The upper helix bundle (UHB) is composed of the central helix and five shorter surrounding helices. The lower helix bundle (LHB) only has three short helices surrounding the central helix (Figure 2E).

Previous studies of HCMV and SCMV particles have suggested pp150 as one of the candidates for the capsid-interacting tegument densities [8], [13], [18]. Secondary structure prediction indicates that the C terminal half of pp150 is almost entirely coils, in contrast to the N terminal half, which contains many helices (Figure 2F). Among these predicted helices, the longest one has 47 residues from a.a.195 to a.a.241 (Figure 2F), which would span ∼70 Å as each amino acid in an α-helix gives an axial distance of 1.5 Å. This ∼70 Å length of the longest predicted helix and the measured ∼67 Å length of the central helix resolved in the cryoEM density correlate well with each other, and both are more than twice the length of any other predicted or resolved helices. Moreover, there are eight other predicted major helices with more than 3 helical turns (each turn = 3.6 a.a.) and their lengths (12–22 a.a.) also correlate with those of the eight shorter helices resolved in the tegument density. Therefore, we conclude that the resolved tegument density is contributed only by the N-terminal half of pp150 molecule, while the C-terminal half of pp150 may be disordered or flexible. This conclusion is consistent with previous biochemical data showing that N-terminal segment of SCMV pp150 was both necessary and sufficient to bind either SCMV or HCMV capsid in vitro [22].

### SCP mediates interactions between pp150 and the capsid

**Figure 3:**
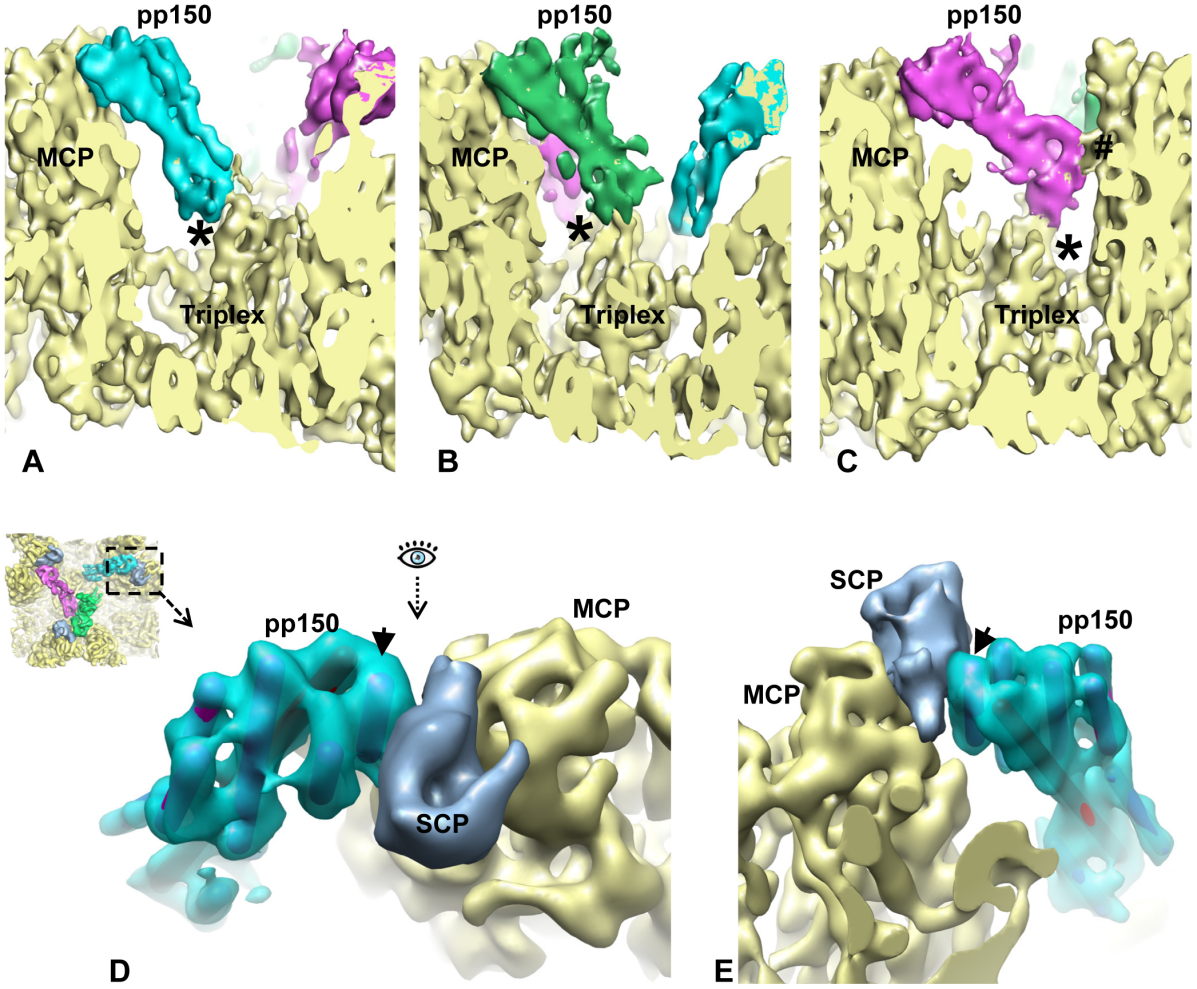
SCP mediates pp150 binding to the capsid. (A–C) Density slices showing that pp150 tegument protein binds to the capsid triplex with its LHB (lower helix bundle). The binding sites on the triplex are labeled with “*”. The LHB of one molecule in the group-of-three tegument densities also has contact with MCP. It is labeled with “#”. (D) A close-up, top view of the region demarcated by the dashed square in the inset, revealing the interactions between pp150 (cyan), SCP (light blue) and MCPud (yellow). (E) Same as in (D) but viewed from the direction indicated by the eye symbol in (D). Arrow in both (D) and (E) points to the α-helix in pp150 UHB (upper helix bundle) that interacts with SCP.

We further identified the interface between the tegument density and the capsid. At one end of pp150, its LHB has direct contacts with the triplex (Figure 3A–C). At the other end, pp150 UHB interacts with the capsomer through one of its five short helices (arrowhead in Figure 3D and 3E). This interaction appears to be mediated by the 8 kDa SCP molecule, which is situated in the cleft formed by the pp150 UHB and the upper domain of MCP (Figure 3D–E). The direct contact between the densities assigned to pp150 and SCP suggests direct binding of the two molecules, although at this resolution, one can't rule out the unlikely possibility that binding of SCP to MCPud can in theory change conformation of MCPud, causing it to bind to pp150 directly.

### Confirmation of the role of SCP by ribozyme inhibition

To assess the functional significance of SCP in mediating pp150 binding to the capsid, we constructed a cell line expressing a ribozyme that inhibits the expression of SCP when the cell line is infected by HCMV. Then, we determined the consequence of this inhibition on pp150-binding to the capsid by cryoEM analyses of viral particles harvested from this cell line.

We constructed a ribozyme, called SCP1, by covalently linking the 3′ terminus of a previously established M1GS ribozyme variant (V482) [23] to an 18-nt guide sequence complementary to the targeted HCMV SCP mRNA sequence. Two other ribozymes, SCP2 and TK1, were also designed and used as controls. SCP2 contains the same guide sequence as SCP1 but has multiple point mutations at the catalytic P4 domain that abolish its catalytic activity [24], thus serving as a control for the antisense effect in our experiments. TK1 targets the mRNA of thymidine kinase (TK) of HSV-1 and serves as a control to determine whether M1GS RNA with an incorrect guide sequence could target SCP mRNA in tissue culture. We subsequently constructed cell lines expressing each of these three M1GS ribozymes and carried out the following three experiments.

**Figure 4:**
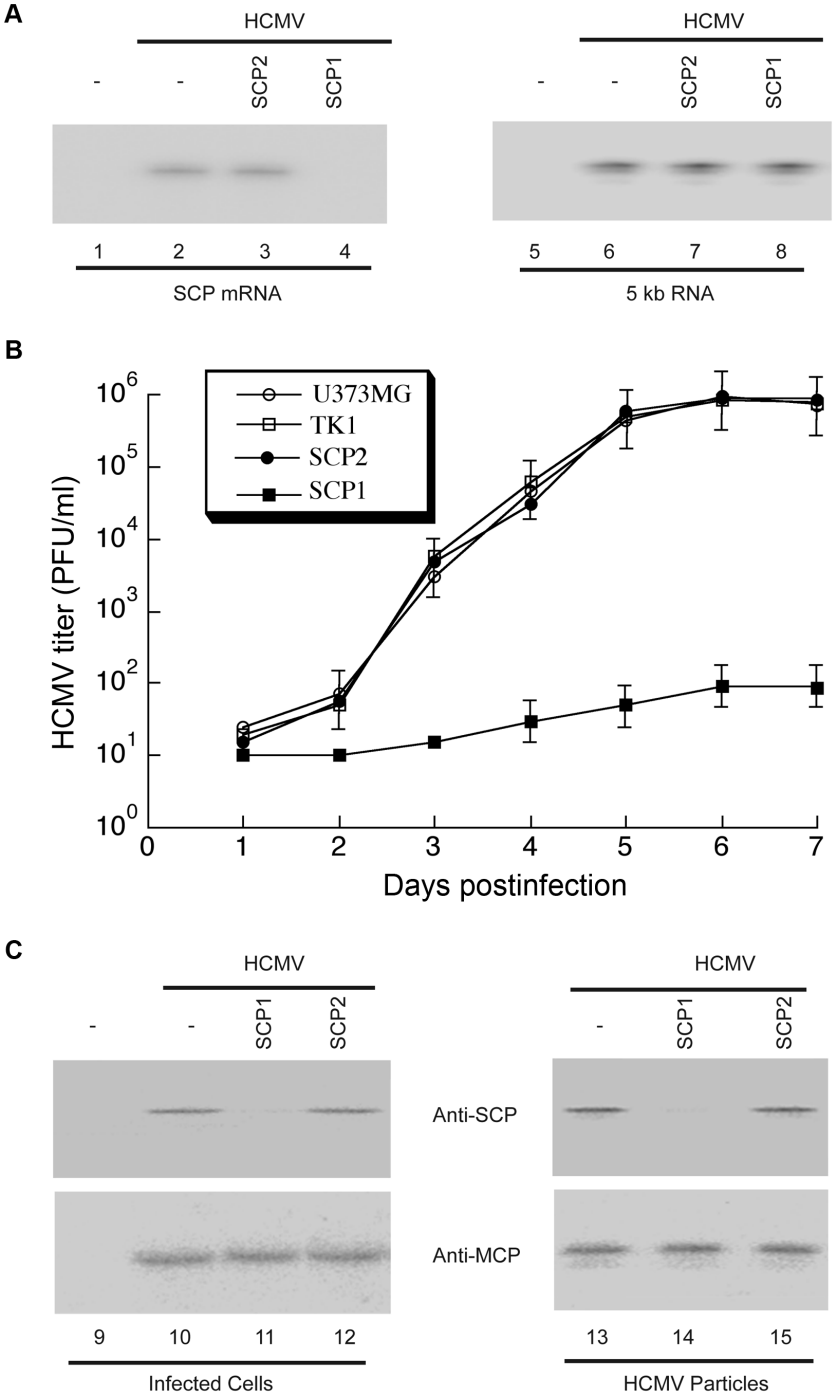
Ribozyme-mediated inhibition of HCMV SCP expression and viral growth. (A) Northern analysis of HCMV mRNAs in infected cells. RNA samples were isolated from parental U373MG cells (lanes 1, 2, 5 and 6) or M1GS-expressing cells (lanes 3, 4, 7 and 8) that were either mock-infected (lanes 1 and 5) or infected with HCMV (MOI = 0.5–1; all other lanes) for 48 h, separated by denaturing gels, and transferred to membranes. Membranes were hybridized with radiolabled probes containing the sequence of HCMV SCP mRNA (lanes 1–4) or IE 5 kb RNA (lanes 5–8). SCP1, ribozyme targeting HCMV SCP mRNA for degradation; SCP2, control ribozyme that binds but cannot degrade HCMV SCP mRNA. (B) Growth of HCMV in U373MG cells and cell lines expressing M1GS RNA. Cells (5×10^5^) were infected with HCMV at MOI = 3. Values are means derived from triplicate experiments. Standard deviation is indicated by error bars. TK1, control ribozyme targeting HSV-1 thymidine kinase mRNA. (C) Western analysis of HCMV SCP and MCP proteins. Protein samples were either isolated from the parental U373MG cells or ribozyme-expressing cells (Infected cells, lanes 9–12) or from viral particle preparations purified from these cells (HCMV particles, lanes 13–15), separated in SDS-polyacrylamide gels, transferred to membranes, and reacted with antibodies against HCMV SCP (anti-SCP) and MCP (anti-MCP) [24].

First, we analyzed SCP mRNA expression in HCMV-infected cells by Northern blotting, using the level of viral immediate-early (IE) 5-kb mRNA as an internal control (Figure 4A). Based on radioactivity of ^32^P-labeled probes, we estimated that target mRNA expression level was reduced by 98±8%, 7±4%, and 3±3% (average of three experiments) in cells expressing SCP1, SCP2 and TK1, respectively. Furthermore, the protein level of SCP, as determined by Western analyses with the MCP protein level as the internal and loading control, was reduced by 97±9%, 8±5%, and 2±1% in cells expressing SCP1, SCP2, and TK1, respectively (Figure 4C, lanes 9–12). Thus, targeted cleavage of SCP mRNA by ribozyme SCP1 significantly reduced SCP expression in cells expressing SCP1, but not in cell lines expressing both control ribozymes. The low level of inhibition observed in SCP2-expressing cells was probably due to an antisense effect, as SCP2 has a target-binding affinity similar to that of SCP1 but is catalytically inactive.

Second, we assessed the effect of SCP-inhibition in viral yield by measuring viral titers of stocks from HCMV-infected cells that express the ribozymes. At 5 days post-infection, viral yields were reduced by at least 10,000-fold in cells expressing SCP1, whereas no significant reduction was observed in cells expressing SCP2 or TK1 (Figure 4B).

**Figure 5:**
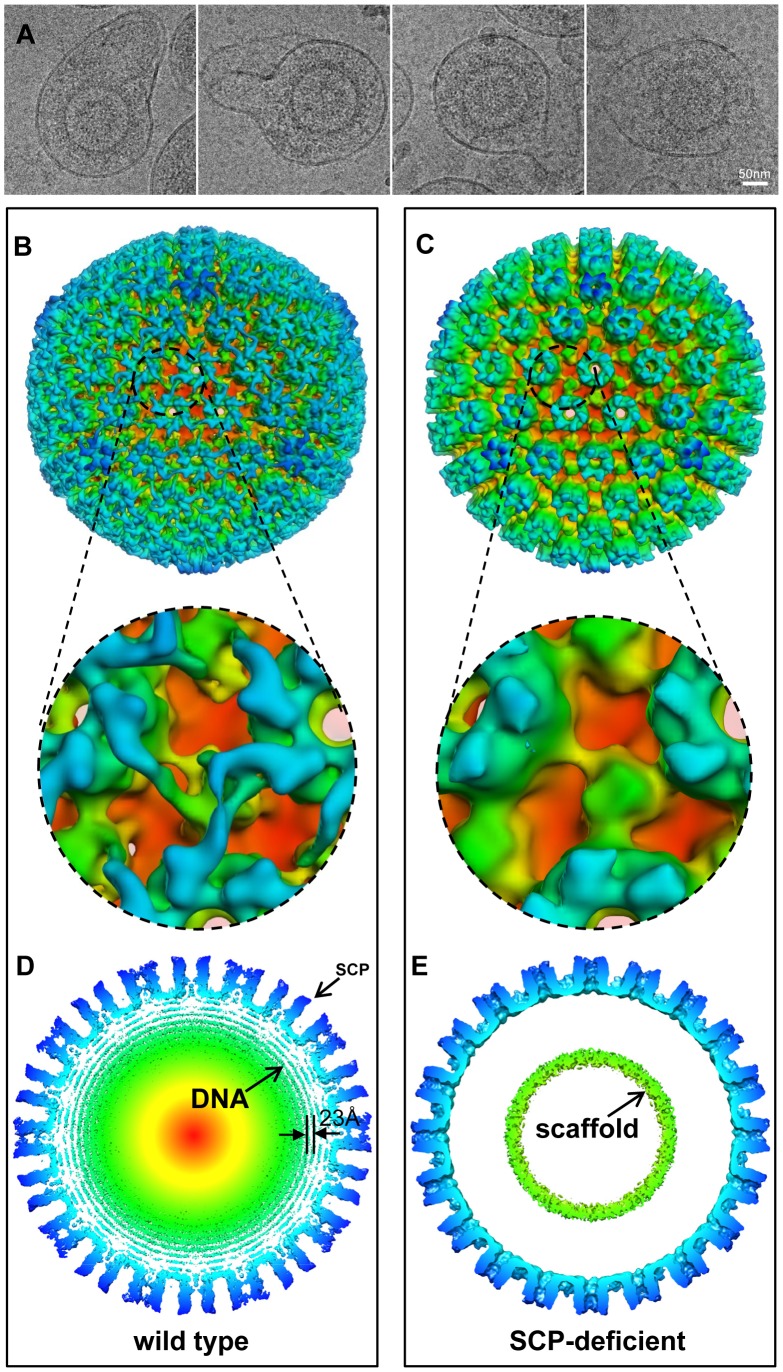
Confirmation of the role of SCP by structural comparison of SCP-deficient and wild-type viral particles. (A) Representative cryoEM images of SCP-deficient viral particles showing enveloped particles without the dsDNA genome. (B, C) Radially colored surface representations of 3D reconstructions of wild type (B) and SCP-deficient (C) HCMV viral particles at 20 Å resolution. Lower panels are zoom-in views of the region containing a triplex, revealing that pp150 is present in the wild-type structure but absent in the SCP-deficient viral particles. (D, E) 15 Å-thick central slices extracted from reconstructions of wild type (D) and SCP-deficient (E) particles respectively. Concentric shells of density inside the capsid in (D) are attributable to the viral dsDNA genome, and they are uniformly spaced (23 Å). A ring of scaffold densities are identified in (E), but there is no DNA density. Small bulge on tip of MCP in (D) corresponds to the density of SCP. There is no such bulge at the corresponding position in the SCP-deficient reconstruction.

Third, to uncover the structural basis of the reduction of viral yield due to SCP inhibition, we imaged viral particles isolated from SCP1-expressing cells by cryoEM and compared its 3D structure with that of the wild-type HCMV virion. Using MCP as the internal and loading control, Western analyses showed that SCP was hardly detected in HCMV particles isolated from SCP1-expression cells but was readily found in viral particles isolated from cells that did not express any ribozymes or expressed control ribozymes SCP2 or TK1 (Figure 4C, lanes 13–15). CryoEM images of wild-type HCMV virion have the characteristic “fingerprint” appearance (Figure 1B), which is a hallmark of encapsidated genomic dsDNA [14], [25]. In contrast, none of the cryoEM images of particles harvested from the SCP1-expressing cell culture media shows a fingerprint pattern (Figure 5A), indicating that they do not contain viral DNA genome and are thus non-infectious. The existence of non-infectious enveloped particles (NIEPs) in this preparation indicates that the inhibition of SCP expression does not prevent capsid assembly and envelopment. Furthermore, 3D reconstruction at 20 Å resolution of these SCP-deficient particles shows a structure with no visible tegument densities bound to the capsid (Figure 5C). In contrast, reconstruction of wild-type virion at the same resolution clearly shows tegument densities interacting with the underlying capsid (Figure 5B). This result clearly demonstrates that SCP is required for the functional binding of pp150 to capsid. Considering that pp150 may function in stabilizing the dsDNA-filled C capsid [17], [18], we reason that, in the absence of SCP, pp150 can no longer form the stabilizing network of density surrounding the capsid, thus preventing the formation of DNA-containing virion (Fig. 5A). However, we cannot rule out the possibility that the lack of DNA in the SCP-deficient particle and failure to bind pp150 are two unrelated, downstream consequences of lacking SCP. It is also noteworthy that, although the absence of SCP prevents pp150 from binding to the capsid with icosahedral symmetry, it does not necessarily eliminate binding of pp150 to the capsid triplex in a non-icosahedrally ordered fashion, which could have also produced a cryoEM map without visible pp150 densities.

## Discussion

As mentioned above, among all human herpesviruses, HCMV has the largest dsDNA genome contained within a capsid of similar size. As a result, the distance between adjacent dsDNA duplex in HCMV capsid is 23 Å [26], as compared to 26 Å and 25 Å for those in alphaherpesvirus [14] and gammaherpesvirus [27], respectively. It is conceivable that the electrostatic repulsion of the more densely packed genome in HCMV would exert higher pressure to the capsid shell, possibly rendering the DNA-containing capsid (i.e., “C capsid”) unstable. Indeed, throughout our cryoEM imaging of the HCMV capsid preparation, not a single intact DNA-containing capsid was observed among the over 30,000 particle images we examined (Figure 1A), in stark contrast to the situations of alphaherpesvirus [25] and gammaherpesvirus [27] where C capsids can be readily purified from the nuclei of infected cells. Upon tegumentation, including the addition of pp150, DNA-containing nucleocapsids are stabilized and thus are routinely found in HCMV virion preparations (Figure 1B).

Of the capsid structural proteins, SCP is the least conserved across different herpesviruses in size, amino acid sequence, and function. For example, the HSV-1 SCP has a molecular weight of 12 kDa and is dispensable for virus growth in cell culture [28]. The 16 kDa KSHV SCP is the largest and is essential for capsid assembly [29], [30]. The 8 kDa HCMV SCP is the smallest and is essential for virus growth [12], but its functional role is a long-standing mystery. Here, by using SCP-targeting ribozyme and cryoEM reconstruction, we provide the first evidence that SCP is required to stabilize DNA-containing HCMV capsids, and that it may do so by directly or indirectly mediating pp150 binding to the capsid. To our best knowledge, this role is the only function of HCMV SCP identified to date. This result, when considered together with the absence of a pp150 homolog in both alpha- and gammaherpesviruses, indicates that SCP of herpesviruses has diverged in function though its location in different herpesviruses is conserved. Perhaps, SCP has a yet unknown, non-essential function conserved across all herpesviruses, but in HCMV, it is re-utilized by pp150 as a partner to stabilize DNA-containing capsid, an essential process for HCMV infection.

Taken into consideration the relatively small size and essential function, HCMV SCP clearly provides a potential target for intervention against HCMV infection. One possible way is to design SCP-mimicking peptides that act as competitive inhibitors of pp150 binding and functioning, thus preventing infectious viral particle formation.

## Materials and Methods

### HCMV virion preparation

Human fibroblast MRC-5 cells were cultured in Dulbecco's Modified Eagle Medium (DMEM) plus 10% fetal bovine serum (FBS). 20 flasks (175 cm^2^) of cells were grown to 90% confluence and infected with HCMV strain AD169 at a multiplicity of infection (MOI) of 0.1–1. At 6 days post infection, when half of the cells were lysed, the media were collected and centrifuged at 10, 000 g for 15 min to remove cell debris. The clarified supernatant was collected and centrifuged at 80, 000 g for 1 hr to pellet HCMV virions. Pellets were resuspended in a total volume of 2 ml phosphate buffered saline (PBS, pH 7.4) and loaded on a 15%–50% (w/v) sucrose density gradient and sedimented at 100,000 g for 1 hr. Usually we observe three light-scattering bands – top, middle and bottom – containing mainly NIEPs, virions and dense bodies, respectively. The middle band was collected and diluted in PBS to a total volume of 13 ml. Virion particles were pelleted again at 80,000 g for 1 hr and resuspended in 30 µl PBS for cryoEM sample preparation.

### HCMV capsid preparation

To obtain HCMV capsids, we infected 90% confluent MRC-5 cells at MOI = 5. At 3 days post infection, when cytopathic effect reached 100%, cells were collected, pelleted by low-speed centrifuge at 1000 g for 10 min, and washed with PBS. The pellet was then resuspended in PBS containing 0.5% NP-40 (w/v) and incubated on ice for 5 min. The mixture was centrifuged at 1000 g for 10 min to pellet cell nuclei. To break nuclear membrane, the pellet was then resuspended in PBS, subjected to three cycles of freezing (−80°C, 10 min), thawing (37°C, 3 min) and vortexing, passed through a 23 gauge hypodermic needle for 20 times, and incubated in PBS with 2% NP-40 overnight at 4°C. The lysate was centrifuged at 1500 g for 5 min to remove large debris and then sedimented through a 30% sucrose cushion at 100,000 g for 1 hr. The pellet was resuspended in PBS containing 2% NP-40, diluted to a final volume of 13 ml, and centrifuged again at 70,000 g for 1 hr. The pelleted capsids were resuspended in 30 µl PBS and used for cryoEM sample preparation.

### Construction and *in vitro* characterization of SCP-targeting and control ribozymes

Plasmids V482, pFL117 and pC102 contain the DNA sequences coding for variant V482 RNA, M1 RNA and mutant C102, respectively, driven by the T7 RNA polymerase promoter [19], [24], [31]. Mutant ribozyme C102 contains several point mutations at the catalytic domain (P4 helix). The DNA sequence coding for ribozyme TK1, which targets the mRNA of thymidine kinase of HSV-1, has been described [31]. The DNA sequence encoding ribozyme SCP1 was constructed by PCR with V482 as the template. The 5′ and 3′ PCR primers were AF25 (5′-GGAATTCTAATACGACTCACTATAG-3′) and M1SCP1 (5′-CCCGCTCGAGAAAAAATGGTGCTGAGCAAGTATACGCGTGTGGAATTGTG-3′), respectively. The DNA sequence coding for ribozyme SCP2 was constructed by introducing into the DNA sequence coding ribozyme SCP1 with the point mutations (A_347_C_348_→C_347_U_348_ and C_353_C_354_C_355_G_356_→G_353_G_354_A_355_U_356_) that were found in C102 and were shown to abolish the ribozyme activity [19], [24], [31]. The procedures for *in vitro* cleavage and binding analyses were carried out as described previously [24].

### Construction of ribozyme-expressing cells

The DNA sequences encoding the ribozymes were subcloned into retroviral vector LXSN and placed under the control of the U6 RNA promoter. The retroviral DNA containing the ribozyme sequence was transfected into human U373MG cells, using protocols modified from Miller and Rosman [32]. After 48–72 h of transfection, cells were incubated in culture medium containing 600 µg/ml neomycin. Cells were subsequently selected in the presence of neomycin for 2 weeks, and neomycin-resistant cells were cloned [24].

### Northern and Western analyses of viral gene expression

Cells (*n* = 1×10^6^) were either mock-infected or infected with HCMV at an MOI of 0.05–5 in 1.5 ml DMEM supplemented with 1% FBS. After 2 h incubation, the inoculum was replaced with DMEM supplemented with 10% (v/v) FBS. The infected cells were incubated for 4–72 h and total cellular RNA or protein was isolated from the cells as described previously [24]. Protein samples were also prepared from HCMV particles purified from the infected cells. The RNA fractions were separated in formaldehyde-containing 1% agarose gels, transferred to a nitrocellulose membrane, hybridized with ^32^P-radiolabeled RNA probes containing the HCMV sequences, and analyzed with a STORM840 PhosphorImager. The RNA probes used to detect M1GS RNA, HCMV IE 5-kb RNA transcript, and SCP mRNA were synthesized from plasmids pFL117, Cig27, and pSCP, respectively [24], [33]. RNA probes were in vitro synthesized and radiolabeled using an in vitro RNA synthesis kit (Promega, Inc, Madison, IN).

In Western analysis experiments, protein samples were separated on SDS/7.5% polyacrylamide gels cross-linked with N,N″methylenebisacylamide, and then transferred electrically to nitrocellulose membranes. We stained the membranes using the antibodies against HCMV proteins in the presence of a chemiluminescent substrate (GE Healthcare), and analyzed the stained membranes with a STORM840 phosphorimager [24]. Quantitation was performed in the linear range of RNA and protein detection.

### Assays for inhibition of viral replication

Cells (*n* = 1×10^5^) were infected with HCMV at MOI values specified in the Results section. The cells and medium were harvested at 1-day intervals throughout the 7 days after infection. Viral stocks were prepared by adding an equal volume of 10% (v/v) skim milk, followed by sonication. The titers of the viral stocks were determined by infecting 1×10^5^ human foreskin fibroblasts and counting the number of plaques 10–14 days after infection [24]. The values obtained were averages from triplicate experiments.

### Purification of SCP-deficient viral particles

Ribozyme SCP1-expressing U373MG cells were infected with wild type HCMV at MOI = 1–5. After 2 hr incubation, the medium was replaced with fresh DMEM plus 10% FBS to remove any free, extracellular viral particles. At 4 days post infection, viral particles were purified using the same procedure as described above for the wild type HCMV virion. Due to the significantly lower viral yield in SCP1-expressing cells, no clear light-scattering bands were visible in the density gradient. We therefore collected the fraction of the gradient corresponding to the range encompassing the three bands visible in the wild type virion purification. This fraction was diluted in PBS and centrifuged at 80,000 g for 1 hr to pellet SCP-deficient viral particles. The pellet was then resuspended in 10 µl PBS, verified by negative staining electron microscopy to contain viral particles, and used for cryoEM sample preparation. The remainder gradient was also collected in fractions of 3 ml each. Each fraction was diluted with PBS, pelleted, resuspended in 10 µl PBS, and checked individually with negative staining electron microscopy to confirm the absence of viral particles.

### CryoEM imaging and data processing

An aliquot of 2.5 µl purified sample was applied to a 300 mesh Quantifoil R1.2/1.3 grid, blotted with filter paper, and plunge-frozen in liquid ethane. CryoEM images were collected at liquid nitrogen temperature in an FEI Titan Krios cryo electron microscope operated at 300 kV with parallel illumination. The wild type HCMV virion and SCP-deficient HCMV particle images were recorded on a Gatan 4k×4k charge-coupled device (CCD) camera at an effective magnification of 97, 498× (nominal magnification 59, 000× on film plane), corresponding to an effective pixel size of 1.538 Å/pixel at the specimen level. The HCMV capsid images were recorded on Kodak SO-163 films at a magnification of 59,000× and micrographs were digitized with Nikon Coolscan 9000ED scanner at a step size of 6.35 µm/pixel, giving a pixel size of 1.076 Å/pixel on specimen. In all cases, the electron dosage used in cryoEM imaging was ∼25e^−^/Å^2^. The defocus values were determined with CTFFIND [34] and are in the range of 0.5 µm to 3 µm underfocus.

Data processing and 3D reconstructions were accomplished with IMIRS [35], [36]. Orientation and center parameters of each particle were refined against projections computed from 3D reconstructions in an iterative procedure until no further improvement in the reconstruction was obtained. Particles were selected based on the phase residues between the images and the projections. 3D reconstruction was obtained using the symmetry-adapted spherical harmonics method [36]. The final capsid and virion reconstructions were obtained by averaging 20,502 particles (selected from 37,460 capsid images) and 11,863 particles (selected from 56,297 virion images), respectively.

Visualization and averaging of density maps were carried out with UCSF Chimera [37]. Density regions to be averaged were segmented out as density cubes of similar size. These density cubes were then first manually aligned and subsequently computationally aligned by the “fit in map” function of Chimera. Averaged density was produced by executing the “vop add” command on the above aligned density cubes.

Secondary structure prediction of pp150 was performed with PSIPRED using the Protein Structure Prediction Server [38].

### Data deposition

The cryoEM density maps of the capsid, the virion, and the SCP-deficient particles have been deposited in the Electron Microscopy Data Bank (EMDB) (accession code 5695, 5696 and 5697, respectively).

## Supporting Information

Figure S1 **FSC plots of HCMV capsid and virion reconstructions.** Based on the FSC = 0.143 criterion, the resolution for the capsid reconstruction is measured to be 6.0 Å and that for the virion reconstruction is 8.3 Å.
(TIF)
