# Supplementary figures and images for: The Smallest Capsid Protein Mediates Binding of the Essential Tegument Protein pp150 to Stabilize DNA-Containing Capsids in Human Cytomegalovirus
Source: PLoS Pathog. 2013 Aug 15;9(8):e1003525. doi: 10.1371/journal.ppat.1003525 (PMC3744435; doi:10.1371/journal.ppat.1003525)

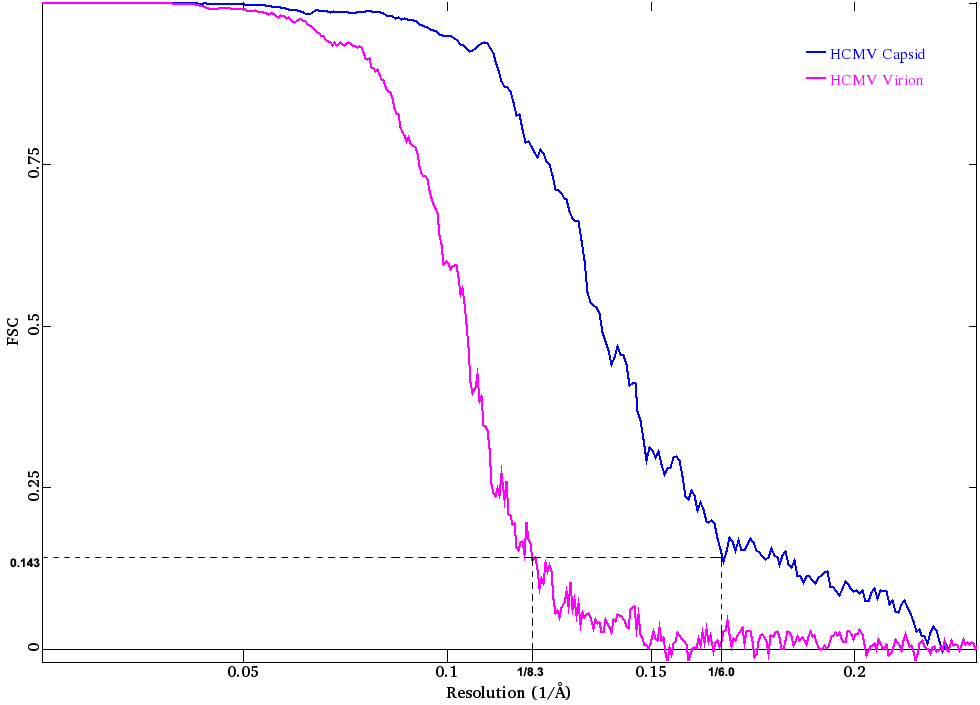

Supplement: Figure S1 — FSC plots of HCMV capsid and virion reconstructions. Based on the FSC = 0.143 criterion, the resolution for the capsid reconstruction is measured to be 6.0 Å and that for the virion reconstruction is 8.3 Å. (TIF) [file ppat.1003525.s001.tif]
